# Comparison between platelet rich fibrin as space filling material versus xenograft and alloplastic bone grafting materials in immediate implant placement: a randomized clinical trial

**DOI:** 10.1186/s12903-023-03678-5

**Published:** 2023-12-08

**Authors:** Heba Abo-Elfetouh Elsheikh, Sally Elsayed Abdelsameaa, Ahmed Adel Elbahnasi, Fakhreldin Hassan Abdel-Rahman

**Affiliations:** 1 Oral and Maxillofacial Surgery, Faculty of Dentistry, Mansoura University, Mansoura, Egypt; 2 Fixed Prosthodontics, Faculty of Dentistry, Mansoura University, Mansoura, Egypt

**Keywords:** Alloplastic bone grafting material, Buccal bone thickness, Immediate implant placement, Marginal bone loss, Platelet Rich Fibrin, Tooth extraction, Xenograft

## Abstract

**Background:**

This study aimed to compare the efficacy of different gap filling materials in immediate implant in anterior and premolar regions of maxilla.

**Materials and methods:**

Thirty-six implants were inserted in patients seeking for replacement of non-restorable maxillary anterior and premolar teeth (esthetic zone) by immediate implant. Patients were randomly distributed into three equal groups, twelve implants in each group. Group 1 received Platelet Rich Fibrin (PRF) into the jumping distance, Group 2 received Xenograft into the jumping distance and Group 3 received Alloplastic bone grafting material into the jumping distance. Implant stability by measuring the changes in Resonance Frequency Analysis (RFA), peri-implant pocket depth, marginal bone loss and changes in buccal bone thickness were evaluated during follow up periods. All the clinical and radiographic data were subjected to statistical analysis by One Way ANOVA test and the Post Hoc Tukey test.

**Results:**

This study involved 19 female patients and 17 male patients who received 36 dental implants. There was no significant difference between the study groups regarding implant stability, peri-implant pocket depth and palatal bone loss, while there was a significant difference between PRF Group (Group 1) and the other Groups regarding buccal bone loss and changes in buccal bone thickness.

**Conclusion:**

PRF can be used as a gap filling material in conjunction with immediate implant placement, but other bone grafting materials give superior result regarding buccal bone loss and changes in buccal bone thickness.

**Trial registration:**

The study was listed on www.clinicaltrials.gov with registration number (NCT05878392) on 26/05/2023. The Institutional Review Board (IRB) of the Faculty of Dentistry, Mansoura University, Mansoura, Egypt, approved the current study in compliance with the seventh revision of the Helsinki Declaration in 2013 (A0103023OS).

## Background

The indications for dental implant treatment options have greatly expanded in recent years due to development in biomaterials and clinical procedures [1]. Dental implants have been successfully used to replace missing teeth, and different insertion and loading protocols have been developed from the original protocol to facilitate quicker and less difficult surgical procedures [2].

Original protocol (gold standard) recommended a 6- to 12-month waiting time before implant placement in cases where a tooth had to be extracted and replaced. New methods that involve implant placement during tooth extraction have been developed. This protocol is called immediate implant placement [3]. Since, the first report of the placement of a Tübinger dental implant into a fresh extraction socket, there has been increasing interest in this technique [2].

Immediate implant placement has an overall survival rate of 98.5%, while delayed implant placement has a survival rate of 98.9% [4]. The possible benefits of immediate implant placement have been suggested to include shorter treatment period, more patient comfort, fewer surgical procedures, optimal three-dimensional implant positioning, preservation of alveolar bone, and better soft tissue contour [5].

In contrast, immediate implant placement has some disadvantages including, lack of control of the final implant position, difficulty of achieving primary stability, incomplete soft tissue closure over the extraction socket, inability to inspect all aspects of the extraction site for infection and difficulty in preparing the osteotomy due to bur movement (chatter) on the walls of the extraction site [6].

After tooth extraction, the alveolar socket frequently has dimensions larger than the implant's diameter, creating a space between the implant's surface and the alveolar bone walls in the recipient site. This area is designated as the jumping distance or the peri-implant gap. The peri-implant gap affects osseointegration and implant stability [7, 8].

The buccal aspect of an implant is of great concern, especially in the aesthetic zone (between the second contralateral premolars), because the buccal bony plate is thin and its resorption can result in soft tissue recession [9, 10], so the peri-implant gap must be filled with bone [11].

Defects < 2 mm can be filled with bone without the need for bone grafts or the usage of barriers. The success of immediate implant procedures may be adversely affected by large gaps, as has been reported [12, 13].

Regarding the best methods to achieve the following goals—optimal bone fill in the gap, the highest level of coronal bone to implant contact (BIC), the least amount of buccal bone resorption, and the least amount of soft tissue recession— the best surgical approach for treating the buccal gap is debatable and unclear [11]. For the treatment of the buccal gap, a variety of methods, such as the use of barrier membranes and grafting materials, have been employed [14].

The peri-implant gap has been filled using several bone grafting materials. Autograft is the gold standard for bone grafting materials but it has some limitations such as the need of a second surgical site, limited volume and size mismatch [14].

Growth factors, such as Platelet Rich Fibrin (PRF) and bone morphogenic proteins, have been used to stimulate bone formation in the defective sites, as has been reported in several studies [15, 16]. In bone augmentation procedures, PRF has been introduced as an additional or replacement material to guide new bone formation. According to Choukroun et al. [17], PRF is a unique technique for concentrating platelets (preparation without thrombin). According to in vitro studies, PRF improves cell proliferation, adhesion, migration and osteogenic differentiation [18]. In addition, PRF inhibits osteoclastogenesis, reduces inflammation, and promotes the expression of many growth factors in mesenchymal cells [19, 20].

The fibrin clot formed during the production of traditional PRF or its modification, is a three-dimensional scaffold that replaces the extracellular matrix in cell regeneration and newly formed vessels. Platelets trapped between fibrin fibers, B and T lymphocytes, monocytes, stem cells and neutrophils, as well as secreted growth factors such as TGF-1, PDGF, and VEGF, play a role in healing [21]. In clinical applications, PRF has been used in the treatment of periodontal defects, sinus floor elevation, and preservation of the alveolar ridge after tooth extraction [19].

The purpose of this study was to compare the efficacy of different gap filling materials in immediate implant in anterior and premolar regions of maxilla. The primary objective was to assess alveolar bone loss and changes in buccal bone thickness radiologically using cone-beam computed tomography (CBCT) and the secondary objective was to assess various clinical parameters such as implant stability by measuring the changes in Resonance Frequency Analysis (RFA) and peri-implant pocket depth.

The hypothesis was that the peri-implant alveolar bone loss, changes in buccal bone thickness and soft tissue health in immediate implants with a jumping distance grafted with PRF would be the same as those grafted with Xenograft or Alloplastic bone grafting materials.

## Materials and methods

### Patient selection

Thirty-six patients, nineteen females and seventeen males with an average age 33 years (range from 19 to 47), were included in this study. They were chosen from the Outpatient Clinic in the Oral and Maxillofacial Surgery Department, Faculty of Dentistry, Mansoura University, Mansoura, Egypt, for replacement of non-restorable maxillary anterior and 1^st^ premolar teeth (esthetic zone) by immediate implant. The Institutional Review Board (IRB) of the Faculty of Dentistry, Mansoura University, Mansoura, Egypt, approved the current study in compliance with the seventh revision of the Helsinki Declaration in 2013 (A0103023OS). The study was following CONSORT guidelines for clinical trials. The study was listed on www.clinicaltrials.gov with registration number (NCT05878392) on 26/05/2023. All of the participants gave their written informed consent. 

### Sample size calculation

The total sample size was determined to be 10 implants in each group using G*power version 3.0.10 to calculate sample size-based t test = 2.31, 2-tailed, α error = 0.05 and power = 90.0% with effect size (2.63). To account for potential attrition by 20%, two additional implants were added to the total sample size (12 implants in each group). This calculation was based on a previous study by Oates and colleagues [22].

### Randomization

**Fig. 1:**
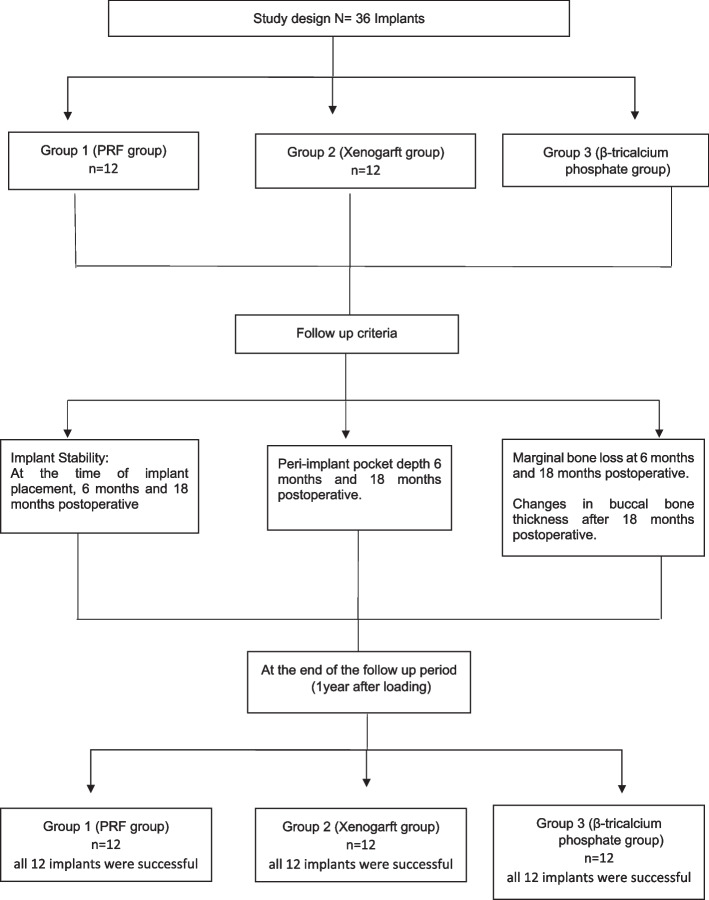
Flowchart representation of groups distribution

One of the department's senior residents, who was not involved in the study and was not aware of any relevant treatment protocols, carried out the randomization. 36 candidates were randomly distributed into three equal groups, 12 implants for each by using a computer-generated randomization list (SPSS v25.0). The distribution of the groups was Group 1 received PRF into the jumping distance, Group 2 received Xenograft into the jumping distance and Group 3 received Alloplastic bone grafting material into the jumping distance. The study design can be seen in Fig. 1.

### Blinding

It was impossible to blind the operator and the operator was not involved in either the distribution or evaluation processes. Furthermore, all patients were unaware of which group they were in. Throughout the follow-up times, the assessor carried out each evaluation step while being entirely unaware of the treatment protocol. Likewise, statisticians were unaware of treatments and groups.

### Criteria for patient selection

**Table:** 

**Inclusion criteria**	**Exclusion Criteria**
1. Patient medically free from systemic diseases	1. A medical condition that would prevent implant surgery
2. Age above 18 years	2. Existence of non-treated generalized progressive periodontitis
3. A single maxillary anterior or 1^st^ premolar tooth that couldn’t be restored	3. Smoker patients
4. Intact socket walls after tooth extraction	
5. No acute infection was present	
6. Jumping gap more than 2 mm in size	
7. Free from history of bruxism	

### Preoperative measures

**Fig. 2:**
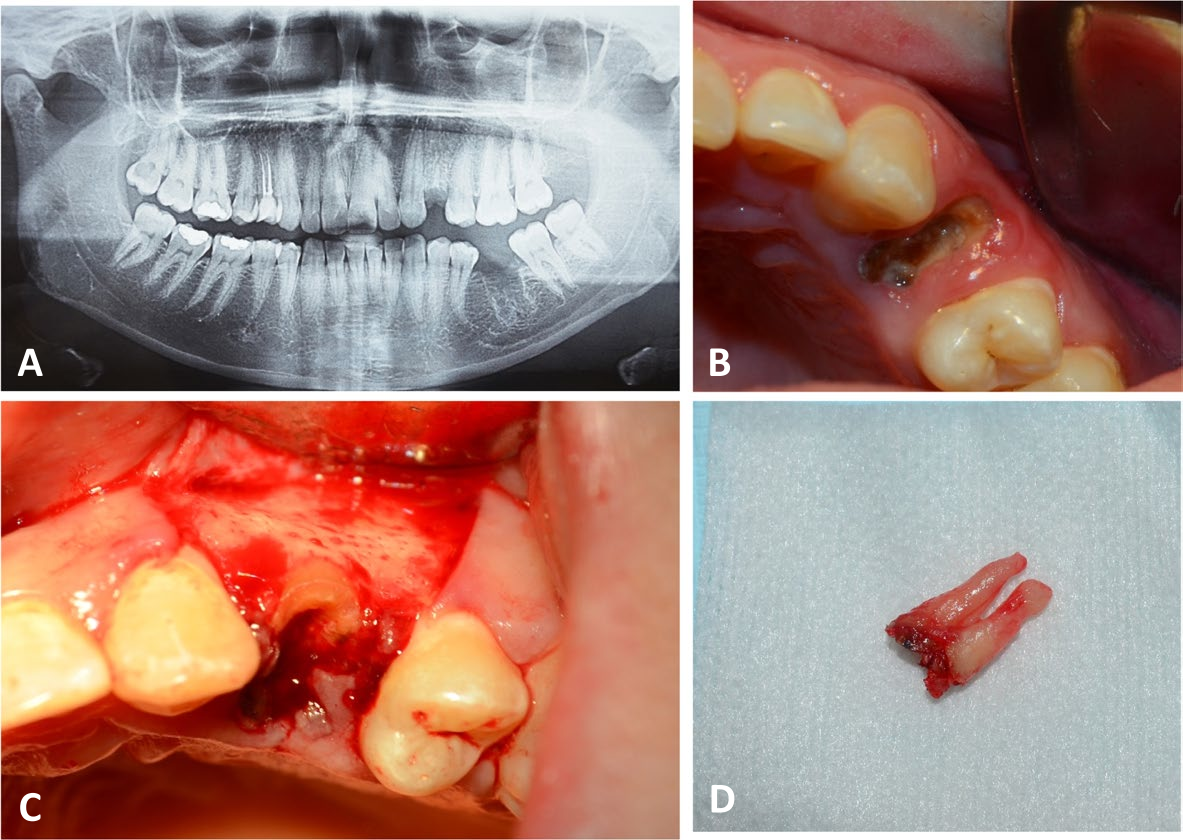
PRF Group **A** A preoperative panoramic radiograph showing badly decayed upper left 1^st^ premolar. **B** A photograph showing badly decayed upper left 1^st^ premolar. **C** Extraction socket after flap reflection. **D **Tooth after atraumatic extraction

For all patients, panoramic radiographs were taken to assess the mesiodistal width, the amount of bone above the apex and the root angulation **(**Figs. 2A and 6A**)**. Two days before surgery, a prophylactic antibiotic regimen of 500 mg of amoxicillin (Emox, Egyption Int. Pharmaceutical Industries Co., E.I.P.I.C.O., A.R.E.) was prescribed every six hours. Before surgery, the patients were rinsed with Chlorohexidine HCl (0.12%) (Hexitol, the Arab Drug Company, Cairo, A.R.E.) for 1 min.

**Fig. 3:**
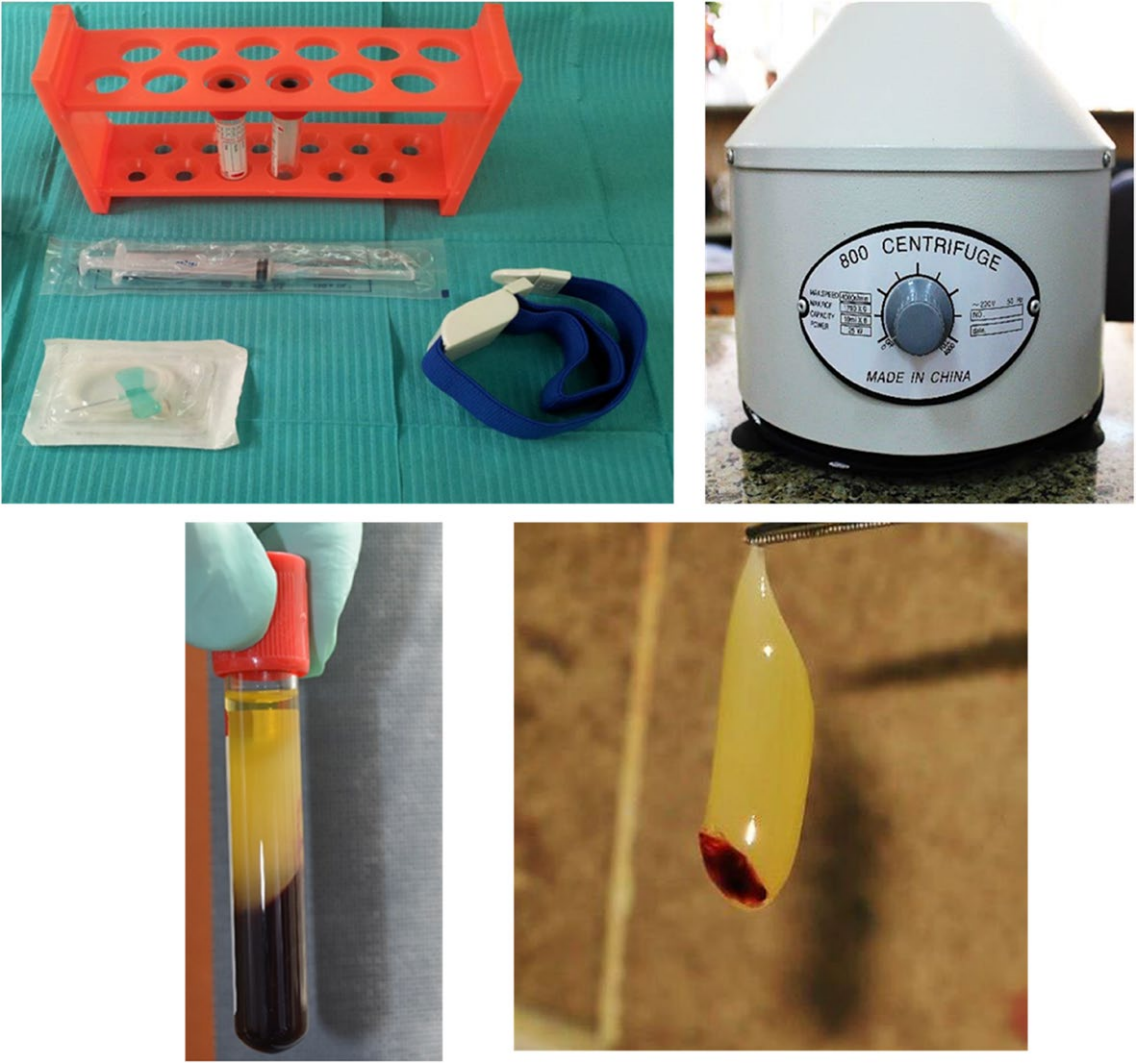
PRF preparation

### Surgical procedures

Following administration of local anesthesia (Mepivacaine HCL 2% with Levonordefrin 1:20,000. Alexandria Co. for Pharmaceuiticals and Chemical Ind., Alexandria, Egypt.), a three-line incision was made, and the mucoperiosteal flap was reflected. Atraumatic extraction of the tooth/root was then initiated by using a periotome (Helmut Zeph, Medizintechnik GMBH, Seitingen-Oberflacht, Germany) to sever the periodontal ligament attachments and preserve the socket walls followed by using suitable extraction forceps (Figs. 2B, C, D and 6B). After tooth extraction, the socket was checked for the integrity of its four walls. If the buccal bone was fractured during the extraction or there was fenestration/dehiscences, the patient was excluded from the study. To remove any granulation tissue that might have been there, the socket underwent a cautious, thorough curettage. For tension-free primary closure, a periosteal release incision was made.

**Fig. 4:**
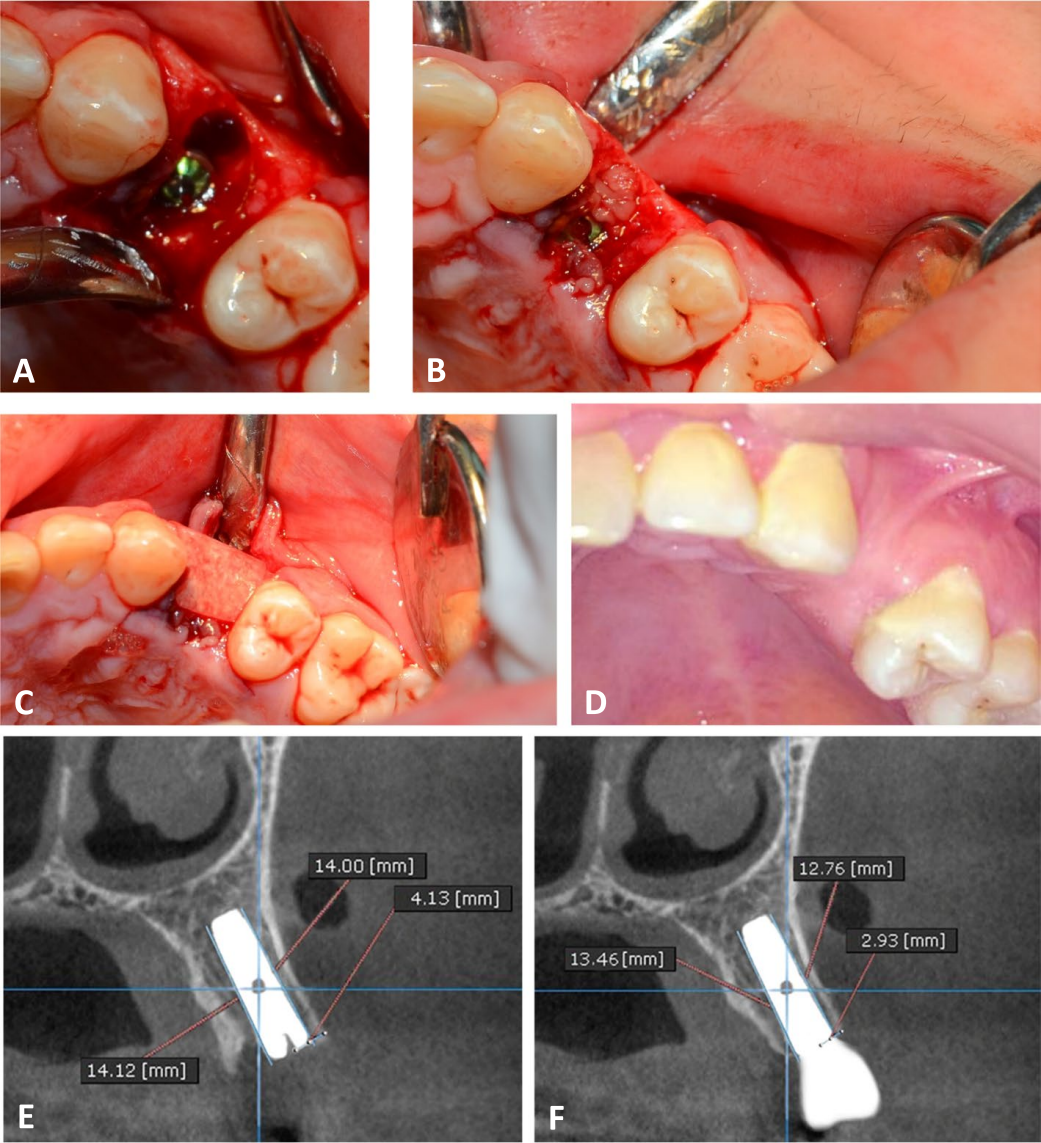
PRF Group. **A** Immediately placed implant with horizontal critical-sized gap. **B** PRF after filling the gap around the dental implant. **C** The collagen membrane after its application. **D** The complete gingival healing after 1 month. **E** An immediate postoperative cross-sectional CBCT image. **F** A cross-sectional CBCT image taken 18 months postoperative

**Fig. 5:**
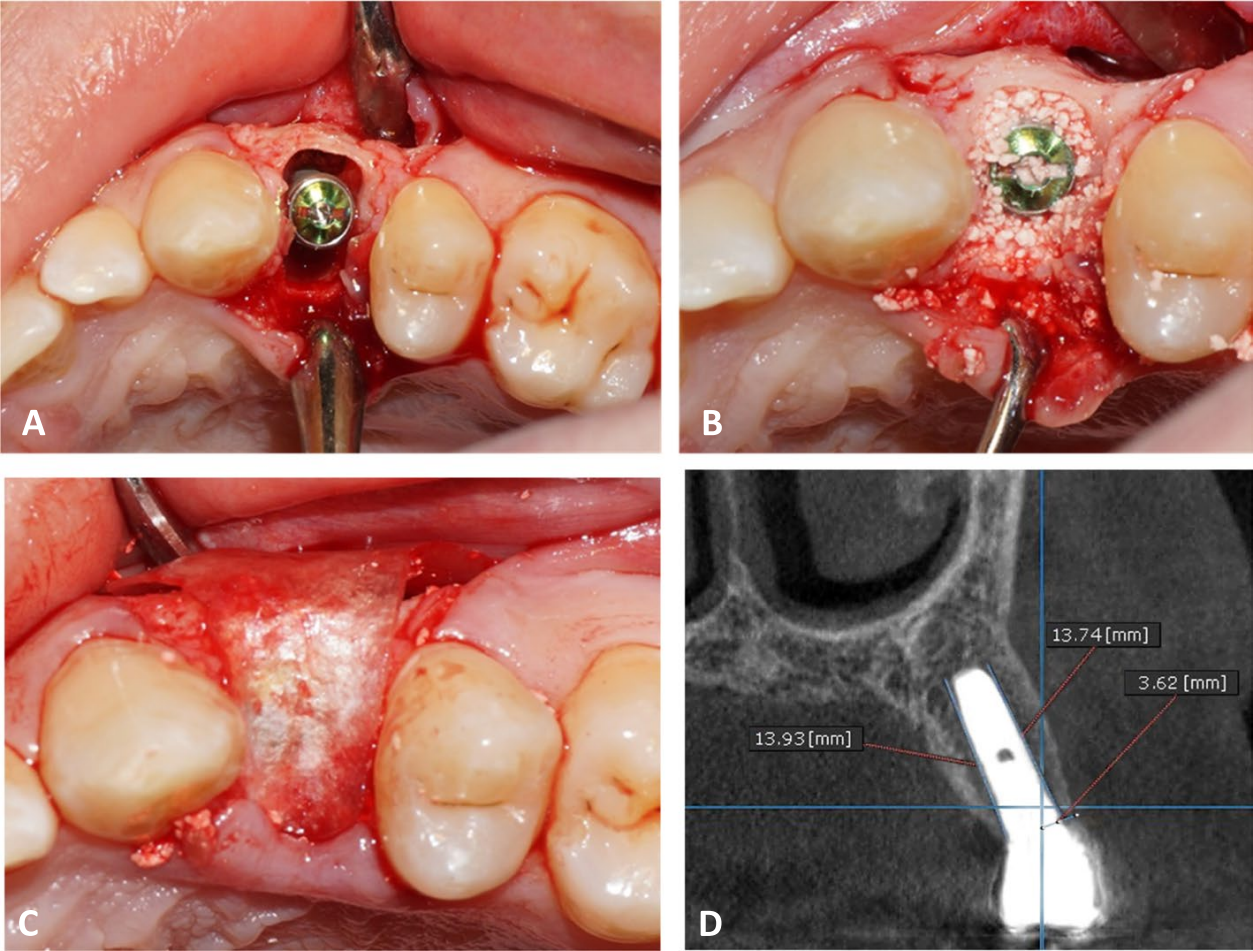
Xenograft Group **A** Palatally positioned dental implant following tooth extraction. **B** Xenograft filling the jumping gap. **C** The collagen membrane after its application over Xenograft. **D** A cross-sectional CBCT image taken 18 months postoperative

The final decision regarding the size of the implant was made after assessing the dimensions of the socket. Drilling was done in the right direction at 600 to 800 rpm. Depending on the implant size, sequential drilling with abundant irrigation was done until the ideal dimensions were achieved. The sterile implant package was opened, and with gentle, steady finger pressure, the implant was placed in its proper location with a manual ratchet (40 Ncm of torque) (Figs. 4A and 5A). In order to achieve primary stability, the implant was installed 2–3 mm beyond the apex and 1–2 mm below the alveolar crestal bone.

The implants used in this study were double-threaded, two-piece, tapered body titanium dental implants with SLA surface. (Dentium® System, Superline, Seoul, Korea.)

After implant placement, the buccal jumping gap was measured using periodontal probe to make sure that the distance from the implant surface and the buccal plat was more than 2 mm.

RFA was used to test implant stability with an Osstell Mentor device. (Osstell, Integration Diagnostics, Savadaled, Sweden). The smart peg (type 7) was attached to the dental implant. The outcomes were presented as the implant stability quotient (ISQ).

The buccal jumping gap in Group 1 was packed using PRF. Platelet-rich fibrin preparation, around 5–10 ml of whole venous blood was collected in each of the two sterile vacutainer tubes without anticoagulant. The vacutainer tubes were then placed in a centrifugal machine (Laboratory Centrifuge, Jiangsu, China) at 3000 rpm (800 gm) for 10 min, after which it settled into the following layers: red lower fraction containing red blood cells, upper straw coloured cellular plasma and the middle fraction containing the fibrin clot. The upper straw coloured layer was then removed and middle fraction was collected, 2 mm below lower dividing line, which was the PRF [23] (Fig. 3). The gap between the walls of the socket and the dental implant was filled with PRF, which was delicately placed and compressed around the implant (Fig. 4B). For Group 2, the buccal jumping gap was packed using Xenograft (Creos xenogain®, Nobel Biocare, Zürich-Flughafen, Switzerland) (Fig. 5B), while for Group 3, the buccal jumping gap was packed using Alloplastic β-tricalcium phosphate (R.T.R Syringe, β-TCP Synthetic granules, Septodont, France.).

**Fig. 6:**
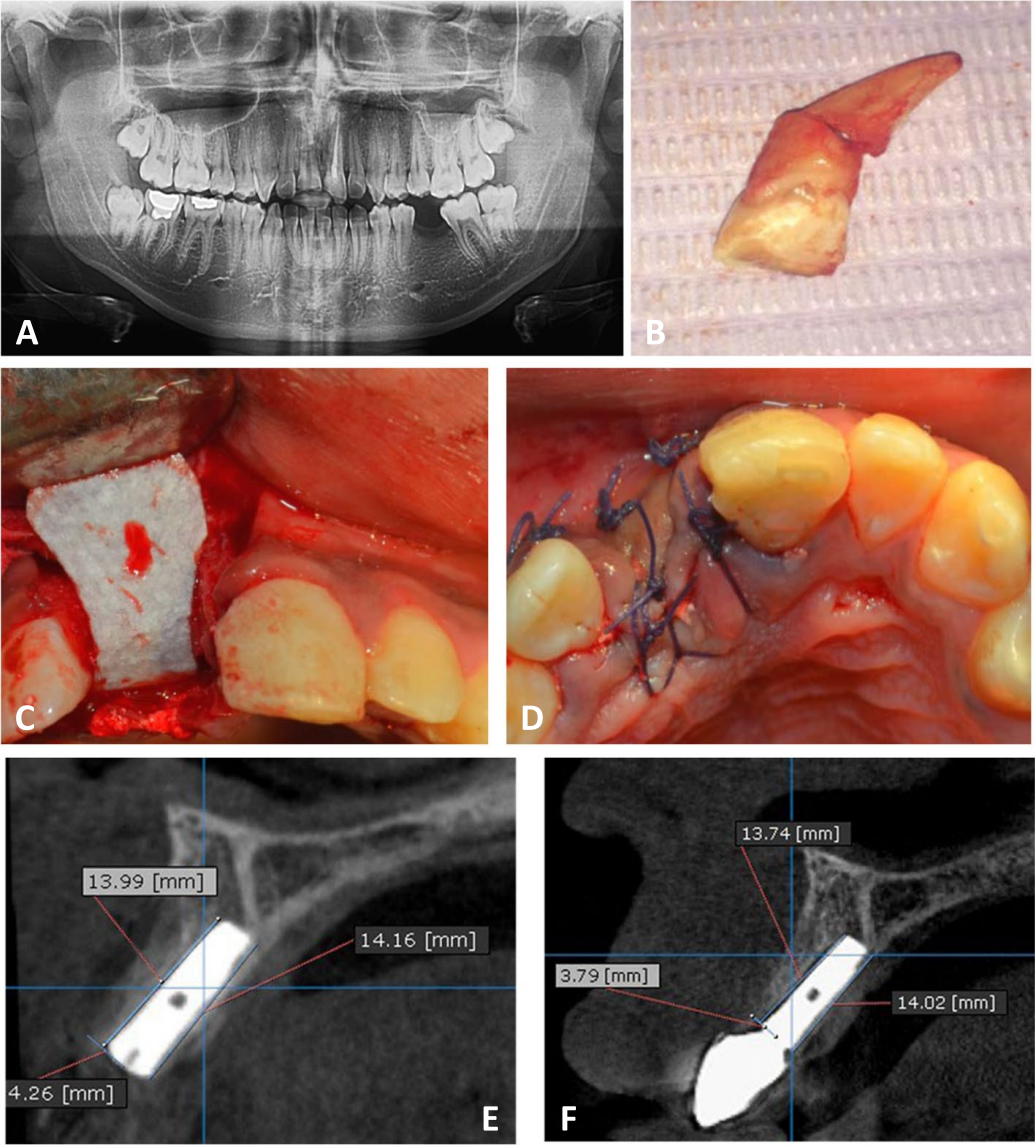
Alloplast Group. **A** The preoperative panoramic radiograph showing horizontal root fracture of upper right central tooth. **B** The tooth after extraction. **C** The collagen membrane after its application. **D** The primary closure of the flap. **E** An immediate postoperative cross-sectional image of CBCT. **F** A cross-sectional CBCT image taken 18 months postoperative.

The next step was to open the sealed package of the collagen membrane (Dentium® System, Resorbable membrane, Korea). The membrane was then trimmed to the size needed by the case. Care was taken to apply the membrane without wrinkling or buckling (Figs. 4C, 5C and 6C). Following the repositioning of the mucoperiosteal flap, the primary closure was completed with both mattress and interrupted 4/0 sutures (Figs. 4D and 6D). CBCT radiograph was taken to verify the final position of the implant.

### Postoperative care

For seven days, 500 mg of Amoxicillin (Emox, Egyption Int. Pharmaceutical Industries Co., E.I.P.I.C.O., A.R.E.) was used as an oral antibiotic every six hours. A non-steriodal analgesic and anti-inflammatory medication called Diclofenac Potassium 50 mg tablets (Oflam, Mepha Pharma Egypt S.A.E.) was prescribed. Patients were advised to avoid chewing solid food, and to maintain good oral hygiene with Chlorohexidine HCl (0.12%) (Hexitol, the Arab Drug Company, Cairo, A.R.E.). Then, after one week, the sutures were removed.

### Second stage surgery

Six months later, a second stage surgery was carried out. The surgical cover screw was exposed and replaced by a healing abutment for 15 days.

### Prosthetic rehabilitation

To create a working cast, an impression was made using an impression post and a laboratory analogue. Then the functional abutment replaced the healing abutment. Final restoration was made from porcelain fused to metal and cemented to the functional abutment.

### Evaluation

Every patient was seen on a regular basis for evaluation immediate, 6 and 18 months postoperative.

#### A. Clinical evaluation

##### 1. Implant stability

At the time of implant placement, 6 months and 18 months postoperative, implant stability was measured. RFA was used to measure implant stability with an Osstell Mentor device. The outcomes were presented as ISQ.

##### 2. Peri-implant pocket depth

A graduated probe was used to measure the distance between the base of the pocket and the gingival margin. The probe was introduced until its blunt edge made contact with the base of the pocket in a straight line with the implant's vertical axis. Around each implant, the pocket depth was measured at 4 different sites (mesial, buccal, distal and palatal). Measurements were taken and recorded to the nearest 0.5 mm.

#### B. Radiographic evaluation

CBCT was used to provide radiographic evaluation immediately, 6, and 18 months postoperative. All CBCT scans were performed in the same radiology centre (Planmeca, ProMax® 3D Max, Helsinki, Finland) using the same parameters (89 kVp, 24 s, 10 mA and field of view 6 cm × 8 cm). For image processing and reconstruction, OnDemand3D was used.

##### 1. Radiographic assessment of marginal bone loss

The implant was utilized as a reference for the measurement of marginal bone loss (MBL) from the cross-sectional view by adjusting panoramic long axis in its center and bisecting it (showing the buccolingual dimensions).

At the crest of the buccal plate of bone and ending at the apical level of the implant, a line was drawn directly parallel to the implant, and its height was measured in millimeters immediately, 6 months and 18 months postoperative. The measurement of the bone level at implant placement was considered as baseline. Radiographic MBL was calculated as the difference between the reading at 6 and 18 months postoperative and the baseline value.^24^ The same process was repeated from the palatal direction (Figs. 4E, F, 5D, 6E and F).

##### 2. Radiographic assessment of changes in buccal bone thickness

A perpendicular horizontal measurement was taken from the implant crest to the buccal bone plate immediately postoperative. This measurement acts as a baseline. A similar measurement was taken 18 months postoperative and subtracted from baseline value to determine horizontal bone loss.^25^ (Fig. 4E, F, 5D, 6E and F).

### Statistical analysis

SPSS software, version 25 was used to analyze the data (SPSS Inc., PASW statistics for windows version 25. Chicago: SPSS Inc.). Quantitative data were described using mean ± standard deviation for normally distributed data after testing normality using Shapiro Wilk test. To compare more than two independent groups, the One Way ANOVA test was performed, and the Post Hoc Tukey test was utilized to identify pairwise comparisons. Significance of the obtained results was judged at the (≤ 0.05) level.

## Results

### Demographic data

This study involved 19 female patients and 17 male patients who received 36 dental implants to replace non-restorable maxillary anterior and premolar teeth (esthetic zone) by immediate implant. The average age was 33 years (range from 19 to 47 years). The distribution of replaced teeth was 20 maxillary central incisor, 8 maxillary lateral incisor, 2 maxillary canine, and 6 maxillary 1^st^ premolar. All patients received porcelain fused to metal crown restorations after 6 months. After 18 months postoperative, all 36 implants in the three groups were successful with 100% survival rate (Table 1).

**Table 1:** Patient’s demographic data

**Patient Grouping**	**Sex**	**Age**	**Tooth no**
**PRF Group**	Female	19	11
	Female	20	21
	Male	33	12
	Female	45	21
	Male	47	11
	Female	28	21
	Female	20	22
	Male	38	11
	Male	39	21
	Female	40	24
	Female	45	21
	Male	44	14
**Xenograft Group**	Female	42	14
	Female	38	12
	Male	35	22
	Female	36	21
	Male	28	11
	Female	26	11
	Male	19	22
	Male	47	11
	Female	33	13
	Female	45	11
	Female	29	24
	Male	30	21
**Alloplast Group**	Male	35	11
	Male	24	12
	Female	29	23
	Female	24	21
	Female	31	14
	Male	33	24
	Male	38	11
	Male	24	21
	Male	47	11
	Male	46	22
	Female	25	22
	Female	29	11

### Comparison of implant stability between the study groups

Implant stability was evaluated using ISQ at surgery, 6 months and 18 months postoperative without statistical difference at surgery (*P* = 0.114), at 6 months (*P* = 0.119), or at 18 months (*P* = 0.216) (Table 2).

**Table 2:** ISQ at different time intervals

**Implant stability**	**Group 1** **(PRF)**	**Group 2 (Xenograft)**	**Group 3** **(Alloplast)**	***P *value**
At surgery	64.33 ± 2.77	65.08 ± 2.27	66.33 ± 2.57	*P* = 0.114
6 months	71.83 ± 2.41	72.35 ± 2.35	73.83 ± 3.16	*P* = 0.119
18 months	73.50 ± 2.07	73.92 ± 2.39	74.92 ± 1.38	*P* = 0.216
	*P* < 0.001*	*P* < 0.001*	*P* < 0.001*	

The *P*-values were calculated by Post -Hoc Tukey test, parameters described as Mean ± SD

*statistically significant

### Evaluation of the peri-implant pocket depth

The peri-implant pocket depth's mean values were all within acceptable ranges (2.5-4 mm). Between the study groups, there was no statistically significant difference at 6 months and at 18 months (*P*-value > 0.05) (Table 3).

**Table 3:** Peri-implant pocket depth at different time intervals

**Peri-Implant Pocket Depth**	**Group 1** **(PRF)**	**Group 2 (Xenograft)**	**Group 3** **(Alloplast)**	***P *value**
6 months	1.54 ± 0.23	1.40 ± 0.25	1.57 ± 0.43	*P* = 0.396
18 months	2.42 ± 0.46	2.32 ± 0.33	2.49 ± 0.27	*P* = 0.533

The *P*-values for peri-implant pocket depth were calculated by Post -Hoc Tukey test, parameters described as Mean ± SD

### Assessment of marginal bone loss

Bone loss changes was evaluated according to each material. Regarding buccal bone loss, there was a significant difference between PRF Group (Group 1) and the other Groups (Group 2 and Group 3) at 6 months and 18 months postoperative (*P* < 0.001) (Table 4).

**Table 4:** MBL at different time intervals

**Buccal Bone Loss**	**Group 1** **(PRF)**	**Group 2 (Xenograft)**	**Group 3** **(Alloplast)**	***P *value**
6 months	0.789 ± 0.15^AB^	0.406 ± 0.12^A^	0.433 ± 0.12^B^	*P *< 0.001*
18 months	1.53 ± 0.095^AB^	0.762 ± 0.12^A^	0.802 ± 0.10^B^	*P* < 0.001*
**Palatal bone loss**	**Group 1** **(PRF)**	**Group 2 (Xenograft)**	**Group 3** **(Alloplast)**	***P*** **value**
6 months	0.401 ± 0.10	0.436 ± 0.15	0.435 ± 0.07	*P* = 0.693
18 months	0.720 ± 0.18	0.779 ± 0.29	0.795 ± 0.29	*P* = 0.636

The *P*-values were calculated by Post -Hoc Tukey test, parameters described as Mean ± SD

Similar superscripted letters denote significant difference between groups within the same row

*statistically significant

Regarding palatal bone loss, there was no significant difference between study groups at 6 months (*P* = 0.693) and at 18 months postoperative (*P* = 0.636). (Table 4).

### Assessment of changes in buccal bone thickness

Bone loss changes was evaluated according to each material. Regarding buccal bone thickness changes, there was a significant difference between PRF Group (Group 1) and the other Groups (Group 2 and Group 3) after 18 months postoperative (*P* < 0.001) (Table 5).

**Table 5:** Changes of buccal bone thickness after 18 months

**Change of buccal bone thickness**	**Group 1** **(PRF)**	**Group2** **(Xenograft)**	**Group3** **(Alloplast)**	***P *value**
	1.56 ± 0.52^AB^	0.65 ± 0.31 ^A^	0.69 ± 0.32^B^	*P* < 0.001*

The *P*-values were calculated by Post -Hoc Tukey test, parameters described as Mean ± SD

Similar superscripted letters denote significant difference between groups within the same row

*statistically significant

## Discussion

Immediate implant placement has highly predictable means of tooth replacement and shows high success rate [26]. However, attaining positive results following immediate implant placement depends on a clear diagnosis and treatment planning. Immediate implant insertion is a sensitive procedure, so certain factors must be carefully taken into account before the procedure is carried out [27].

The area from the upper 1^st^ premolar to the maxillary tuberosity is considered posterior region from a prosthetic point, but anatomically, the bone quality in the upper 1^st^ premolar area is similar to the canine region than the molar region [28]. In our study, non-restorable maxillary teeth from right first premolar to left first premolar were selected to be replaced with immediate implant placement.

Before implant placement, the dimensions of the socket must be evaluated to establish the length and diameter of the implant. To obtain primary stability, the drilling extended 3–4 mm apically to engage the bone beyond the apex of the extraction socket [13]. Additionally, this ensures that the implant will be positioned 1–2 mm below the alveolar crest to achieve an appropriate bone level at the time of implant exposure and to maintain a favorable prosthetic position [29].

An essential step in preventing infection and epithelial downgrowth at the implant site is primary flap closure [30]. A periosteal releasing incision was made, and a flap was cronally adjusted to achieve primary closure.

The implant diameter should be smaller than the socket width, and the implant should be positioned palatally to ensure a minimum horizontal distance of 2 mm between the implant crest and the buccal bone to prevent buccal bone resorption [25]. This causes a gap between the implant's cervical region and the bone tissue as well. Small gaps heal naturally without the need for repair [13, 31], but it is recommended to use bone graft if the buccal gap is more than 2 mm horizontally [32].

Concentrated platelets have been utilized in wound healing in recent years due to their high growth factor concentration [33]. A typical platelet concentration called PRF is made up of an autologous leukocyte-platelet-rich fibrin matrix with a three-dimensional structure that contains cytokines, platelets, and stem cells [34]. These cytokines have significant defense capacities against infections. The large amount of white blood cells has played an important role in anti-inflammatory and anti-bacterial action during the process of immune regulation and angiogenesis [35]. However, few reports have been made about using PRF alone as a filling material for peri-implant bone defects.

Furthermore, PRF can be prepared with antibiotic loading, and the drug is subsequently released from PRF with an antimicrobial effect over four days which can be used after surgical procedures. More in vitro and in vivo studies are needed to prove that PRF loaded with antibiotics represents a topical antibiotic delivery tool for oral surgical procedures that promotes tissue healing and prevents local infection [36].

Implant stability assessment was done using ISQ values of RFA, and there was no significant difference between the three groups at the time of implant placement (*P* = 0.114), at 6 months (*P* = 0.0119) and at 18 months (*P* = 0.216). Primary implant stability was achieved by engaging the palatal wall and the bone approximately 2 mm beyond the apex of the extraction socket. There was a significant difference when comparing implant stability immediately postoperative and at different time intervals in the same group (P < 0.001). This is due to secondary and tertiary stability establishment.

Po-Sung Fu et.al [37] reported that an increase in the mean ISQ over time for all implants which were placed immediately with and without provisionalization Rowan et al. [38] found similar results in his study.

The measurement of the peri-implant pocket depth is (PPD) essential for diagnosing the periodontium. During assessment of PPD, there was no statistically significant difference (*P*-value > 0.05) in all time intervals between the three groups. The peri-implant pocket depth's values were all within acceptable ranges (2.5–4 mm) in the three groups.

Our results were in line with that of Viswambaran et al. [3], whom found the PPD values increased from 6 to 12 months postoperative, but the values within the acceptable range.

In our study, an increase in PPD values can be attributable to reflection of a full thickness mucoperiosteal flap, which results in a junctional epithelium that is more apically positioned. In addition, open wounds heal slowly and with noticeable scarring because the peri-implant mucosa's vascular structure is impaired [39].

A successful implant should have average bone loss of less than 1.5 mm over the first year after loading and less than 0.2 mm annually when measuring MBL [40]. More buccal bone loss was observed in PRF group in comparison to other groups, this result indicates that augmenting the jumping gap with Xenograft or Alloplastic β-tricalcium phosphate led to a better coronal position of the alveolar bone crest than the PRF group. However, clinically PRF resulted in acceptable crestal bone levels which didn’t exceed 1.5 mm after the first year of implant placement.

Our results were in line with that of Elbrashy et al. [25], whom found that more crestal bone loss in a group grafted with PRF than a group grafted with bovine bone.

Regarding buccal bone thickness changes, more reduction in buccal bone thickness in PRF group in comparison to other groups.

These findings are in line with those of Nevins and colleagues [41], who grafted fresh extraction sockets with deproteinized bovine bone mineral (DBBM) and noted that the buccal wall thickness of the grafted sites was slightly reduced in comparison to non-grafted sites. According to Sanz and colleagues [42], the reduction in the horizontal ridge's overall dimension was often less pronounced in grafted sites than it was in non-grafted ones.

This might be explained by the biology of various grafting materials and their rates of resorption. According to various studies, PRF can continue to release growth factors for up to 10 days [43] which is thought to be insufficient period of time to have an impact on the bone remodeling process following extraction and implant placement, which typically lasts for up to 6 months. On the other hand, Xenograft and Alloplastic β-tricalcium phosphate have slow resorption rate and provide a scaffold through which osteoblast cells can impregnate and regenerate bone in the jumping space [44].

The limitations of this study are a small sample size, a relatively short follow up period, and absence of a control group. In addition, soft tissue parameters such as keratinized mucosa width, gingival biotype, and gingival zenith position were not assessed.

## Conclusion

This study demonstrated that the use of Xenograft and Alloplastic β-tricalcium phosphate as filling materials in conjunction with immediate implant have superior results regarding buccal bone loss and buccal bone thickness over use of PRF as a filling material.

## Data Availability

The data sets used and/or analyzed during the current study are available from the corresponding author on reasonable request.

## Abbreviations

BIC: Bone to implant contact
PRF: Platelet Rich Fibrin
CBCT: Cone-beam computed tomography
RFA: Resonance Frequency Analysis
IRB: The Institutional Review Board
ISQ: Implant stability quotient
MBL: Marginal bone loss
PPD: Peri-implant pocket depth
DBBM: Deproteinized bovine bone mineral
